# School and class-level variations and patterns of physical activity: a multilevel analysis of Danish high school students

**DOI:** 10.1186/s12889-018-5155-9

**Published:** 2018-02-14

**Authors:** Carina Bjørnskov Steenholt, Veronica Sofie Clara Pisinger, Ida Høgstedt Danquah, Janne Schurmann Tolstrup

**Affiliations:** National Institute of Public Health, University of Southern Denmark, Studiestræde 6, 1455 Copenhagen K, Denmark

**Keywords:** Physical activity, Cluster analysis, Intra-class correlation coefficient, School environment, Cross-sectional study, Young people, Active transport

## Abstract

**Background:**

There is limited knowledge of physical activity (PA) patterns among high school students. High schools plays an important role as context for the students, but it is uncertain to what extent schools influence student participation in PA during leisure time. The purpose of this study is to describe patterns of PA and assess variations between schools and classes in PA, in a large cohort of Danish high school students.

**Methods:**

Self-reported cross-sectional data came from The Danish National Youth Study, comprising a total of 70,674 students attending 119 different schools and 3213 classes. Multilevel logistic regressions were applied to evaluate the association between socio-demographic variables and patterns of PA, and to assess the impact of schools and classes on PA measures.

**Results:**

Students whose parents have achieved a lower level of education, older students and girls of perceived ethnic minority generally participated less in several forms of PA during leisure time. Substantial variations between schools were observed in terms of participation in PA at school during leisure time and in terms of use of active transportation to and from school. The school-level accounted for 9% (intra-class correlation coefficient (ICC) = 0.09 (95% CI: 0.06–0.11)) and 8% (ICC = 0.08 (95% CI: 0.07–0.11)) of the variation for participation in PA during leisure time and active transportation.

**Conclusion:**

Overall, students whose parents achieved a lower level of education, older students and girls of perceived ethnic minority represent vulnerable groups in relation to participation in several forms of PA during leisure time. The ICCs indicate that schools, in particular, have the potential to influence participation in PA at school during leisure time and active transportation to and from school. Thus, high schools should encourage and facilitate activities aimed at engaging students in PA during leisure time as well as encourage active transportation.

## Background

Physical inactivity is a major independent risk factor for the development of several lifestyle-related diseases and for global mortality [1–4]. The burden of non-communicable diseases on populations’ health necessitates the identification and understanding of physical activity (PA) levels and trends, enabling the development and implementation of effective PA-promoting programmes. Global estimations of PA indicate that nearly a third of individuals, aged 15 years or older, do not comply with recommendations [5]. There is limited knowledge of PA patterns among young individuals (aged 15–25 years) compared to those among adults and children [5].

The high school period is a time of transition characterised by psychological, biological and social changes [6, 7]. Focus on and knowledge of the consequences of these changes is important, as the effect on health behaviours and patterns of declining PA trajectories, which persist into adulthood, might be established during adolescence and young adulthood [8–10]. Gender differences in PA patterns are observed already during childhood, with girls being less active than boys, and inactivity patterns increasing with age [5, 8]. These findings are also observed in the Health Behaviour in School-aged Children (HBSC) study [8, 11] and in objectively measured PA in school children [12, 13]. However, the causes of these differences and to what extent they persist in high school students remains to be elucidated.

High school is an important context for the students, as they spend a large part of their day interacting with the school environment and with other students. It is uncertain to what extent the school influences PA behaviour in this age group, but the school might be an important determinant of the development of PA patterns. In children and adolescents, studies that have assessed the impact of school related factors on PA, have reported large variations in the PA that could be accounted for by schools [14–21]. Except for two [18, 20], these studies were conducted among students attending schools primarily located in the same geographical area or municipality; thus, the impact of the school environment might not be fully reflected in these data. School-level variances might be higher when several municipalities are included, due to greater variations in environmental factors and health policies between schools.

Assessments of different measures of PA participation and the variation between schools and classes have, to our knowledge, not been conducted in a larger sample of high school students. Thus, the purpose of this study is to describe patterns of PA and assess variations between schools and classes in measures of PA, in a national cohort of Danish high school students.

## Methods

### Study population

**Fig. 1:**
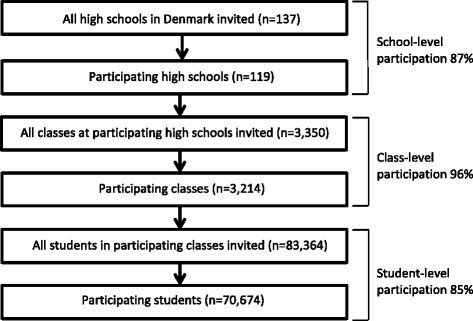
Flow of participating high schools, classes and students in the Danish National Youth Study 2014

Data came from The Danish National Youth Study, which is a national survey conducted among 75,853 high school and vocational school students in 2014 in Denmark. A thorough description of the study is reported elsewhere [22]. In brief, the overall aim was to investigate health, health behaviour and well-being among students in secondary education in Denmark and thus the questionnaire was not designed and implemented specifically to generate data for the students’ PA behaviour. All 137 general high schools and 12 of the largest vocational schools in Denmark were invited to participate. Data was collected from September to December 2014. Teachers provided passwords for the students to access the questionnaire. Students answered the electronic questionnaire in class during one to two lessons of 45 min each. The present study included high school students only. In total, 119 of the 137 general high schools in Denmark participated (87%). All students from participating high schools were invited of whom 85% participated, resulting in a total study population of 70,674 respondents (Fig. 1).

Ethics approval was not necessary under Danish law as this study did not include human biological material [23]. In 2014 The Danish Data Protection Agency approved the study and that all national confidentiality and privacy requirements were met (J. No. 2013–54-0526). Coordinators at the schools gave oral information about the study and the written information made available to the students clearly stated that participation in the study was voluntary and that responses would be treated confidentially. By participating they gave consent that their data could be used for research.

### Questionnaire

The PA outcomes in this paper are based on answers from four items, originating from several sources, which were tailored specifically to the adolescent target group.

The question of assessing physical inactivity during leisure time was based on previously validated measures originally constructed and evaluated by Saltin and Grimby [24, 25]. The variable ‘very active during leisure time’ was assessed using a previously validated question originating from the HBSC study [8, 11]. The questions assessing active transport, organised, self-organised and at-school PA during leisure time was specifically developed for the Danish National Youth Study 2014, in close collaboration with Danish researchers in the field.

Two high schools (1661 students) initially participated in pilot testing of the questionnaire and in focus group interviews, after which small adjustments in the questionnaire were made. However, the items pertaining to physical inactivity during leisure time, organised PA, self-organised PA and PA at school during leisure time were added after the pilot testing. Thus, participants from the pilot schools present missing answers on these items. The items related to organised PA, self-organised PA and PA at school during leisure time were added to obtain information on where students are physically active, which was not covered by the original items related to PA. The construct of each question is elaborated as follows.

#### Inactive during leisure time

Physical inactivity during leisure time was assessed by the question: ‘Within the last year how would you most accurately describe your physical activity during leisure time?’. The answers were dichotomised into ‘Yes’ (inactive) for answers ‘reading, watching television or having other sedentary behaviour’ and ‘No’ for answers ‘exercises vigorously and perform competitive sports regularly several times a week’, ‘practices recreational sports or similar for at least 4 hours a week’, and ‘walks, cycles or performs other light intensity exercise at least 4 hours per week (also including walking and cycling/walking to school)’.

#### Very active during leisure time

To assess the proportion of students who were very physically active during leisure time the following question was used: ‘Outside of school: how many hours a week do you practice sport or other physical activity to the extent that you become breathless or sweat (not including low intensity cycling)?’. The answers were dichotomised into ‘Yes’ for the following answers: ‘about 4–6 h a week’ and ‘about 7 hours a week or more’ and ‘No’ for the answers ‘none’, ‘about half an hour a week’, ‘about an hour a week’, and ‘about 2–3 h a week’.

#### Active transportation

Active transportation to and from school was assessed by the question: ‘How often do you cycle or walk to and from school?’. The answers were dichotomised into ‘Yes’ for answers ‘always’ and ‘for the most part’, and ‘No’ for answers ‘sometimes’, ‘seldom’ and ‘never’.

#### Organised, self-organised or at-school physical activity during leisure time

Participation in organised PA, self-organised PA, or participation in PA at school during leisure time was assessed by the question: ‘Where do you perform sports and other forms of physical activity?’. Participation in organised PA was defined as having answered ‘at the high school’ or ‘in a sports association or club (e.g. football, dance, martial arts)’. Participation in self-organised PA was defined as having answered ‘in a fitness centre (e.g. resistance training, group fitness classes)’ or ‘on your own or self-arranged (e.g. running, swimming, football in the park)’, while participation in PA at school during leisure time was defined as having answered ‘at the high school (not including compulsory physical education lessons)’. As students had the opportunity to tick off more than one answer in this question, the variables organised PA, self-organised PA and PA at school during leisure time were not mutually exclusive.

#### School-level factors

Principals at each high school answered a questionnaire containing questions about school conditions and strategies for improving health and well-being among students. Six school-level factors related to special efforts or projects (1 factor), school facilities (4 factors) and possibilities of PA during leisure time at the school (1 factor) were included from different questions from the principal questionnaire. Whether the school had made a special effort or participated in PA projects was defined as having answered ‘the school has made a special effort or participated in projects with physical activity within the last three years’. Principals were also asked ‘How do you agree or disagree with the following statements about the gymnasium facilities and resources?’ with the possible items: ‘the school has a gym hall suitable for sports’, ‘the school has a varied selection of sports equipment (e.g. balls and fitness machines)’, ‘the school has good changing and shower facilities’ and ‘the school has good access to outdoor football fields (e.g. football, basketball and multi-purpose field/court)’. Responses to these questions were dichotomised into ‘Yes’ for ‘agree’ and ‘No’ for ‘neither agree nor disagree’ and ‘disagree’. To assess whether the school offered PA or sport outside of school hours the following question was used: ‘Does the high school offer any of the following voluntary activities outside normal school hours?’ with one of the possible items ‘sport (e.g. ball games, yoga, resistance training, running)’. Answers to this item were dichotomised into ‘Yes’ for ‘yes, every day’ and ‘yes, at least once a week’ and ‘No’ for the answers ‘yes, less often than once a week’ and ‘no, never’.

The degree of urbanisation, classified into ‘densely populated area’, ‘intermediate populated density area’, and ‘thinly populated area’ was coded according to the EUROSTAT DEGURBA variable according to the location of the schools [26].

#### Student-level factors

Self-reported student-level factors included sex (boy/girl), birthday and year (age in years calculated), perceived ethnicity (Danish/Danish and other/other than Danish), and cohabitation status (living with both parents/living with one parent/other).

Parental educational level (basic schooling/vocational or high school/higher education) was defined by the parents’ highest achieved educational level and was obtained by linkage to the Danish Population Education Register [27, 28].

### Statistical analyses

All analyses were performed with available data, thus ignoring missing data on respective items. To account for dependency among students within the same class and school, multilevel logistic regressions nesting students within school classes (*n* = 3213) within high schools (*n* = 119) were applied to evaluate the association between the independent variables and the PA outcomes. The analyses were sex and age adjusted and presented as odds ratios (OR) with 95% confidence intervals (CI). The variation between school and class was calculated and represented by an intra-class correlation coefficient (ICC). Furthermore, ICC’s for PA at school during leisure time was calculated separately with the inclusion of school-level factors from the principal questionnaire. A nested log likelihood ratio test was conducted to test whether the inclusion of the school-level factors influenced the model.

To determine whether the relation between age and the PA outcomes differed for boys and girls, logistic regressions were conducted including an interaction term (sex*age categories). A log likelihood ratio test was used to analyse if a model including the interaction was better at describing data than a model without interaction. All statistical analyses were performed using Stata version 14.2.

## Results

### Study population

The study population comprised a total of 70,674 high school students who attended 119 different schools and 3213 classes. Overall, the mean age of the students was 18 years and there were more girls (61%) than boys (39%) participating. Most students characterised themselves as Danish (90%), lived with both parents (64%) and had parents with a higher level of education (61%). Finally, the largest proportions of the schools were located in areas classified as intermediate populated density areas (41%)(Table 1).

**Table 1:** Descriptive characteristics of high school students from the Danish National Youth Study 2014

	Total	Boys	Girls
	*n (%)*	*n (%)*	*n (%)*
Study population	70,674 (100)	27,732 (39)	42,942 (61)
Age, mean years (SD)	18 (1.6)	18 (2.0)	18 (1.3)
School grade			
1st grade	26,410 (37)	10,336 (37)	16,074 (37)
2nd grade	24,551 (35)	9673 (35)	14,878 (35)
3rd grade	19,713 (28)	7723 (28)	11,990 (28)
Ethnicity			
Danish	62,647 (90)	24,059 (89)	38,588 (91)
Danish and other	4853 (7.0)	2064 (7.6)	2789 (6.6)
Other than Danish	2075 (3.0)	1013 (3.7)	1062 (2.5)
Parents’ educational level			
Basic schooling	2803 (4.2)	909 (3.5)	1894 (4.7)
Vocational or high school	22,859 (35)	7923 (31)	14,936 (37)
Higher education	40,603 (61)	17,009 (66)	23,594 (58)
Cohabitation			
Living with both parents	44,935 (64)	18,033 (66)	26,902 (63)
Living with one parent	19,832 (28)	7381 (27)	12,451 (29)
Other	5412 (7.7)	2095 (7.6)	3317 (7.8)
Degree of urbanisation			
Densely populated area	19,793 (28)	8080 (29)	11,713 (27)
Intermediate populated density area	28,856 (41)	11,427 (41)	17,429 (41)
Thinly populated area	22,025 (31)	8225 (30)	13,800 (32)

*Abbreviations: SD* standard deviation

Data are presented as proportions (%) except for age, which is presented as mean (SD). *n* does not sum up to the total study population for all variables due to missing answers on some items

### Measures of physical activity among boys and girls

Not living with both parents or living in thinly populated areas were associated with higher odds for being inactive during leisure time, while having parents with a high level of education was associated with lower odds of being inactive during leisure time. The most pronounced association was observed for ethnicity, where girls who characterised themselves as Danish and other (OR = 2.17 [95% CI: 1.95–2.42]), or other than Danish (OR = 3.38 [95% CI: 2.90–3.95]) had higher odds of being inactive during leisure time as compared to those who characterised themselves as Danish (Table 2).

**Table 2:** Odds ratios (OR) with 95% confidence interval (CI) for measures of physical activity among boys and girls

	Inactive during leisure time *n = 6941 (10%)*		Very active during leisure time *n = 27,484 (40%)*		Organised physical activity *n = 27,865 (44%)*		Self-organised physical activity *n = 53,018 (83%)*		PA at school during leisure time *n = 8223 (13%)*		Active transportation *n = 39,422 (57%)*	
	*Boys n = 2588 (10%)*	*Girls n = 4353 (11%)*	*Boys n = 14,013 (52%)*	*Girls n = 13,471 (32%)*	*Boys n = 13,061 (53%)*	*Girls n = 14,804 (38%)*	*Boys n = 20,110 (81%)*	*Girls n = 32,908 (84%)*	*Boys n = 4158 (17%)*	*Girls n = 4065 (10%)*	*Boys n = 16,171 (60%)*	*Girls n = 23,251 (55%)*
	*OR (95% CI)*	*OR (95% CI)*	*OR (95% CI)*	*OR (95% CI)*	*OR (95% CI)*	*OR (95% CI)*	*OR (95% CI)*	*OR (95% CI)*	*OR (95% CI)*	*OR (95% CI)*	*OR (95% CI)*	*OR (95% CI)*
Age	1.02 (1.00–1.04)	1.06 (1.03–1.08)	0.99 (0.97–1.00)	0.99 (0.97–1.01)	0.98 (0.97–1.00)	0.96 (0.94–0.98)	1.06 (1.03–1.09)	1.13 (1.10–1.16)	1.05 (1.03–1.06)	1.05 (1.03–1.08)	0.93 (0.91–0.95)	0.93 (0.91–0.95)
Ethnicity												
Danish	1.0 (Reference)	1.0 (Reference)	1.0 (Reference)	1.0 (Reference)	1.0 (Reference)	1.0 (Reference)	1.0 (Reference)	1.0 (Reference)	1.0 (Reference)	1.0 (Reference)	1.0 (Reference)	1.0 (Reference)
Danish and other	1.21 (1.03–1.41)	2.17 (1.95–2.42)	0.97 (0.88–1.07)	0.72 (0.66–0.79)	0.87 (0.78–0.97)	0.79 (0.72–0.87)	1.09 (0.96–1.25)	0.90 (0.80–1.00)	1.07 (0.94–1.23)	1.38 (1.21–1.58)	0.81 (0.73–0.89)	0.84 (0.77–0.91)
Other than Danish	1.32 (1.07–1.63)	3.38 (2.90–3.95)	1.00 (0.86–1.15)	0.64 (0.54–0.74)	0.75 (0.65–0.87)	0.63 (0.54–0.74)	0.93 (0.78–1.12)	0.71 (0.60–0.84)	0.87 (0.71–1.08)	1.40 (1.13–1.74)	0.83 (0.71–0.96)	0.82 (0.72–0.94)
Parents’ educational level												
Basic schooling	1.0 (Reference)	1.0 (Reference)	1.0 (Reference)	1.0 (Reference)	1.0 (Reference)	1.0 (Reference)	1.0 (Reference)	1.0 (Reference)	1.0 (Reference)	1.0 (Reference)	1.0 (Reference)	1.0 (Reference)
Vocational or high school	0.76 (0.62–0.94)	0.75 (0.66–0.87)	1.11 (0.96–1.29)	1.26 (1.13–1.41)	1.09 (0.93–1.28)	1.23 (1.09–1.38)	1.05 (0.87–1.27)	1.04 (0.91–1.20)	1.11 (0.89–1.39)	0.77 (0.66–0.91)	1.12 (0.96–1.30)	1.01 (0.91–1.12)
Higher education	0.63 (0.51–0.78)	0.55 (0.48–0.63)	1.00 (0.86–1.16)	1.27 (1.13–1.42)	1.12 (0.96–1.31)	1.33 (1.18–1.49)	1.14 (0.95–1.38)	1.14 (1.00–1.31)	1.21 (0.97–1.50)	0.74 (0.63–0.87)	1.56 (1.34–1.81)	1.38 (1.25–1.53)
Cohabitation												
Both parents	1.0 (Reference)	1.0 (Reference)	1.0 (Reference)	1.0 (Reference)	1.0 (Reference)	1.0 (Reference)	1.0 (Reference)	1.0 (Reference)	1.0 (Reference)	1.0 (Reference)	1.0 (Reference)	1.0 (Reference)
One parent	1.27 (1.15–1.39)	1.19 (1.11–1.28)	0.84 (0.79–0.89)	0.82 (0.78–0.86)	0.76 (0.71–0.81)	0.74 (0.71–0.78)	1.03 (0.95–1.11)	1.07 (1.01–1.14)	1.00 (0.92–1.08)	1.06 (0.98–1.15)	1.02 (0.96–1.08)	1.14 (1.09–1.19)
Other	1.35 (1.16–1.58)	1.34 (1.19–1.51)	0.85 (0.76–0.94)	0.78 (0.71–0.85)	0.83 (0.75–0.93)	0.73 (0.66–0.79)	0.82 (0.72–0.94)	0.90 (0.80–1.00)	1.21 (1.06–1.39)	1.47 (1.30–1.66)	1.39 (1.24–1.55)	1.62 (1.49–1.76)
Degree of urbanisation												
Densely populated area	1.0 (Reference)	1.0 (Reference)	1.0 (Reference)	1.0 (Reference)	1.0 (Reference)	1.0 (Reference)	1.0 (Reference)	1.0 (Reference)	1.0 (Reference)	1.0 (Reference)	1.0 (Reference)	1.0 (Reference)
Intermediate density area	1.13 (0.96–1.32)	1.04 (0.87–1.24)	1.12 (0.96–1.31)	1.17 (1.00–1.37)	1.23 (1.04–1.44)	1.24 (1.04–1.47)	0.94 (0.83–1.06)	0.99 (0.88–1.12)	1.23 (0.93–1.61)	1.09 (0.83–1.45)	0.72 (0.57–0.92)	0.85 (0.68–1.08)
Thinly populated area	1.28 (1.09–1.51)	1.19 (0.99–1.43)	1.11 (0.95–1.30)	1.17 (1.00–1.37)	1.51 (1.28–1.78)	1.67 (1.40–1.99)	0.81 (0.72–0.92)	0.83 (0.74–0.94)	1.45 (1.10–1.90)	1.34 (1.01–1.77)	0.50 (0.39–0.65)	0.58 (0.46–0.74)

*Abbreviations:* CI: confidence interval, PA: physical activity

All variables are age adjusted. *n* denotes the proportion of boys and girls classified within each measure of physical activity

Boys were generally more likely to be very active during leisure time compared to girls (52% vs 32%) and boys and girls not living with both parents were less likely to be very active during leisure time. The proportion of boys and girls who participated in organised PA was 53% and 38%, while 81% and 84% engaged in self-organised PA. Living with ‘other than parents’ and attending a school in a thinly populated area were associated with lower odds of participating in self-organised PA. Whereas attending a school in less densely populated areas and having parents with a high level of education was associated with higher odds of participating in organised PA.

The proportion of boys and girls participating in PA at school during leisure time was 17% and 10%. Higher odds for participating in PA at school during leisure time were observed for girls who characterised themselves as Danish and other or other than Danish, for boys and girls living with ‘other’ than their parents, and attending a school in a thinly populated area. Lower odds for participating in PA at school during leisure time were observed for girls with parents with higher education. For example, girls whose parents’ educational level was higher education, had 0.74 (95% CI: 0.63–0.87) lower odds of participating in PA at school during leisure time, compared to girls whose parents’ educational level was basic schooling.

The proportion of boys and girls using active transportation (i.e. walking or cycling) to and from school was 60% and 55%. Higher parental educational level and not living with parents were associated with higher odds of using active transportation, whereas those characterising themselves as Danish and other, or other than Danish, students of a higher age and those attending a school with a lower degree of urbanisation were associated with lower odds of using active transportation. For example, boys attending schools located in thinly populated areas had 0.50 (95% CI: 0.39–0.65) lower odds of using active transportation, compared to boys attending schools in densely populated areas.

### Measures of physical activity by sex and age

**Fig. 2:**
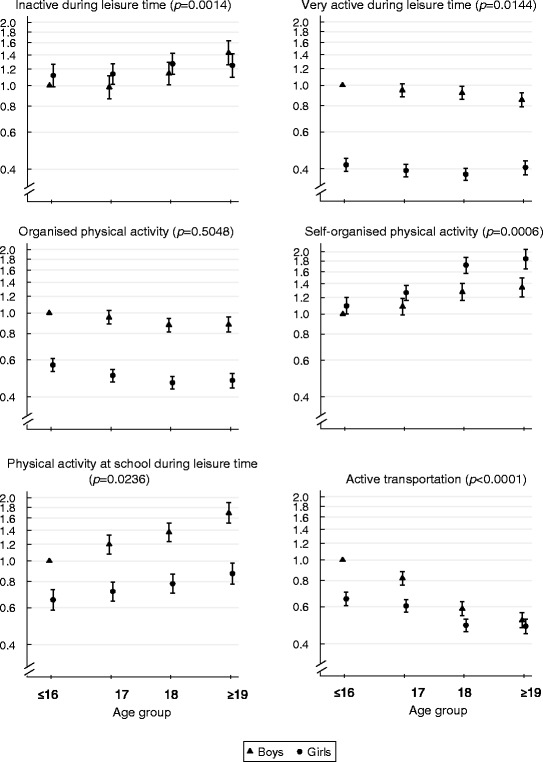
Odds ratios (OR) with 95% confidence interval (CI) for measures of physical activity by age groups among boys and girls. The age groups on the x-axis are ≤16 years (*n* = 14,313), 17 years (*n* = 23,614), 18 years (*n* = 21,313) and ≥19 years (*n* = 11,434) and the reference group is ≤16 year old boys. *P*-values represent the log likelihood test for the model including the interaction between sex and age category compared to the model without

An interaction between sex and the different age categories (≤16 years, 17 years, 18 years and ≥19 years) was found for the following measures of PA: inactive during leisure time (*p =* 0.0014), very active during leisure time (*p =* 0.0144)*,* self-organised PA (*p =* 0.0006)*,* PA at school during leisure time (*p =* 0.0236) and active transportation (*p* < 0.0001). No interaction was observed for participating in organised PA (*p* = 0.5048)(Fig. 2).

The odds of being very active during leisure time and participating in organised PA, PA at school during leisure time and active transportation were generally lower for girls compared to boys in all age groups. Lower odds within the higher age bracket were observed for both sexes for active transportation, while higher odds were observed for girls across age groups for self-organised PA. For example, compared to ≤16 year old boys, the odds of using active transportation were lower for 17 year old boys (0.82 [95% CI: 0.76–0.88]), 18 year old boys (0.59 [95% CI: 0.54–0.63]) and ≥19 year old boys (0.52 [95% CI: 0.48–0.56]).

Furthermore, there was a tendency towards higher odds within the higher age bracket for being inactive during leisure time and participating in PA at school during leisure time and lower odds within higher age bracket for very active during leisure time and organised PA. For example, compared to ≤16 year old boys, the odds of being inactive during leisure time were higher for 18 year old boys (1.14 [95% CI: 1.01–1.29]) and ≥19 year old boys (1.43 [95% CI: 1.25–1.63]).

### School and class-level intra-class correlation coefficients (ICC)

The ICCs between students from the same class ranged from 0.02 to 0.06 and school-level ICCs ranged from 0.01 to 0.09, with the highest ICC observed for PA at school during leisure time. In total, 4–15% of the variation in the PA measures could be attributed to the difference between school and class-level factors (Table 3).

**Table 3:** Intra-class correlation coefficient (ICC) and 95% confidence interval (CI) for measures of physical activity

	*School*		*Class*		*School + Class*	
	*ICC*	*95% CI*	*ICC*	*95% CI*	*ICC*	*95% CI*
Inactive during leisure time (*n* = 67,417)	0.03	(0.02–0.04)	0.06	(0.05–0.06)	0.08	(0.07–0.09)
Very active during leisure time (n = 69,121)	0.03	(0.02–0.04)	0.06	(0.05–0.06)	0.09	(0.08–0.10)
Organised physical activity (*n* = 63,986)	0.04	(0.03–0.06)	0.06	(0.06–0.06)	0.10	(0.09–0.12)
Self-organised physical activity (n = 63,986)	0.01	(0.01–0.02)	0.02	(0.02–0.03)	0.04	(0.03–0.04)
Physically active at school during leisure time (n = 63,986)	0.09	(0.06–0.11)	0.06	(0.06–0.06)	0.15	(0.12–0.17)
Active transportation (n = 69,070)	0.08	(0.07–0.11)	0.04	(0.04–0.04)	0.12	(0.10–0.14)

*Abbreviations: CI* confidence interval, *ICC* Intra-class correlation coefficient, *PA* physical activity

*Inactive during leisure time*, *organised PA*, *self-organised PA and PA at school during leisure time* contain answers from students from 117 schools and 3147 classes. Answers for *very active during leisure time* and *active transportation* are provided by students from 119 schools and 3213 classes

All variables are age and sex adjusted

Consistently lower ICCs were observed after inclusion of the school-level factors, although only special effort or projects in PA (*p* = 0.0309) and PA offered outside of school hours (*p* = 0.0294) significantly influenced the model (Table 4). For example, 72% of the schools offer PA outside of school hours, and after inclusion of this factor the combined school and class-level ICC changed from 0.15 (95% CI: 0.13–0.17) to 0.10 (95% CI: 0.07–0.15).

**Table 4:** Intra-class correlation coefficient (ICC) with 95% confidence intervals (CI) for school-level factors and physical activity at school during leisure time

	*Schools with the facility/initiative*	*School*	*Class + school*
	*n (%)*	*ICC (95% CI)*	*ICC (95% CI)*
Special effort or projects in physical activity^a^	79 (71%)	0.05 (0.03–0.09)	0.11 (0.09–0.15)
Gym hall suitable for sports^b^	102 (88%)	0.05 (0.02–0.13)	0.12 (0.08–0.17)
Good selection of sports equipment^c^	105 (90%)	0.04 (0.01–0.14)	0.11 (0.07–0.17)
Good changing and shower facilities^c^	85 (73%)	0.04 (0.02–0.08)	0.11 (0.08–0.14)
Good access to outdoor football fields^c^	101 (86%)	0.08 (0.03–0.15)	0.14 (0.09–0.20)
Offers physical activity outside of school hours^d^	109 (72%)	0.04 (0.01–0.11)	0.10 (0.07–0.15)

*Abbreviations: CI* confidence interval, *ICC* Intra-class correlation coefficient

All variables are adjusted for age and sex. *n* denotes the proportion of schools reporting having or providing the school facility or initiative

^a^contain answers from 99 schools with students in 2655 classes

^b^contain answers from 114 schools with students in 3064 classes

^c^contain answers from 115 schools with students in 3104 classes

^d^contain answers from 113 schools with students in 3055 classes

## Discussion

This study described patterns of PA and quantified the variations of measures of PA between schools and classes among 70,674 students attending high school in Denmark.

Our findings in relation to PA patterns were consistent with findings in school children and adolescents showing that ethnic minority [20, 29, 30], female sex [5, 8, 12–14, 19–21, 30, 31], higher age [8, 19] and indicators of low parental socio-economic status [8, 13, 14, 20] were negatively associated with PA levels or the likelihood of participating in organised PA. Although a low proportion of students participated in PA at school during leisure time (13%), this finding is encouraging as girls of perceived ethnic minority simultaneously had higher odds of participating in PA at school. This finding could indicate that facilitation of opportunities of PA at the schools during leisure time could potentially activate this otherwise inactive group of girls. This is further emphasised by the finding that 9% of the variance in this outcome was accounted for by school characteristics. After the separate controlling for factors relating to school leadership, we found an attenuation of the ICC for PA during leisure time, suggesting that at least some of the identified school-level variance is due to facilities or opportunities offered by the schools. In support, secondary schools that offer daily PA and have high quality and accessible PA facilities have been found to be predictive for Canadian students’ time spent on moderate to vigorous PA [20, 21]. In this study, perceived ethnic minority, having parents with lower education, higher age and lower school urbanisation degree were independently associated with lower likelihoods of walking or cycling to and from school. This is consistent with active commuting studies in children and adolescents that have shown that ethnic minority [32], female sex [33–36], indicators of low parental socio-economic status [32, 34], higher age [37], longer distance between school and home [33, 37, 38] and lower residential densities [36] negatively influence the use of active transportation to and from school. It is reasonable to assume that students attending schools located in intermediate or thinly populated areas generally have to travel longer distances to school, which explains why fewer students actively commute to school. The emerging pattern of students of a higher age being less likely to actively commute is discouraging, but is presumed to be indicative of students acquiring their driving licence (possible when turning 18 years old) and thus, using passive forms of transport. We found that 8% of the variance in active transport could be explained by school factors, implying that schools have the potential to influence students’ behavioural patterns in relation to active travel. School promotion of active transportation in Canada has been positively associated with higher PA levels in children [19]. Thus, by encouraging and facilitating initiatives of walking and cycling to school in this group of young individuals, the school has the potential to positively influence students’ modes of active travel.

High school-level ICCs indicate that student PA behaviour is influenced by mutually shared social and environmental circumstances at the schools. To our knowledge only Kristensen et al. (2013) has reported ICCs for the separate and combined effect of both the school and the class in children. They showed that classes had a consistently larger impact on PA (accounting for 6–14%), emphasising the importance of psychosocial factors rather than physical conditions of the schools. In line with this, consistently higher ICCs for school classes were observed in most of our PA measures, except for PA at school during leisure time and active transportation where school-level ICCs were higher. This is not that surprising, since students attending the same school are exposed to the same conditions and offered the same possibilities of participating in PA during leisure time at the school regardless of class relations. Like suggested by Kristensen et al. (2013), the higher class ICCs could indicate that circumstances within a class to a large extent influence PA patterns. However, it remains to be elucidated which are the specific class-related psychosocial factors that cause the clustering of these PA behaviours in this age group.

Compared to other health-related behaviours [14, 15, 18] school-level ICCs for PA behaviours are inconsistent and tend to be less than 0.07 [14–21], suggesting little influence on PA behaviour by school characteristics. Although some of our school-level ICCs were low, they were in fact significant for all PA measures, and comparable to that observed in a nationally representative study in secondary school students in England [18] and Canada [21]. The between school variance of 9% and 8% for PA at school during leisure time and active transportation are noteworthy and suggest that schools can influence these behaviours. However, the small between school variance for inactive, very active, organised PA and self-organised PA, suggests that these outcomes are mainly individually determined and that only a small part of these PA characteristics is shared among students at the same school. This is perhaps not surprising as the outcomes address leisure time PA, which might not noticeably be influenced by school factors. Also, the low ICCs could potentially be explained by the fact that high schools in Denmark are largely governmentally administered, ensuring a certain degree of homogeneity. The student composition at the schools and the PA opportunities that they offer are probably, to a large degree quite similar.

A major strength of this study is the sample size and number of schools and classes included, which allowed us to assess separate associations for boys and girls and clustering effects for several measures of PA behaviour. With 70,674 students attending 119 different schools and 3213 school classes, this is to our knowledge the largest survey in relation to young individual’s PA patterns in Denmark and one of the largest globally. In 2014 there were 106,268 students enrolled in Danish high schools [39], of which about 67% were included in this study. Thus, the study population is assumed to represent students in Danish high schools, as student (85%), school (87%) and class (96%) participation were high. Not only is this study nationally representative, the extensive dataset, containing a number of PA questions, permitted detailed description of several patterns of PA participation in this age group.

Some limitations should be noted. Data are based on self-reported questionnaires, which tend to overestimate PA [40, 41]. However, we tried to minimise the overestimation in the outcomes by coding PA variables conservatively and by describing patterns of PA participation instead of levels or intensities of PA. The study is also limited by its cross-sectional design, as conclusions on cause-effect relations cannot be made. Longitudinal investigations are required in assessing the development of PA patterns over time and whether changes in school environmental factors precede change in PA in high school students.

Residual confounding cannot be dismissed. Few studies are available on school-level clustering effects in PA addressing specific factors that could account for the data clustering. Student demographics such as ethnicity, socio-economics and gender have shown to reduce school-level ICCs [14, 16, 18]. PA clustering effects have also proven not to be influenced by adjustments for gender, socio-economic status, parental exercise habits, grade, year, and season of measurement [17]. Likewise, we did not find that ICCs decreased in size for our outcomes when adjusting for socio-demographic variables, which suggest that the observed school-level variance is related to unidentified factors that are beyond the scope of this paper. It is possible that school-level effects in high school may reflect residual school effects from primary school, since students often transition together from primary to high school.

Our results contribute new and important knowledge about PA patterns and the impact of schools in young individuals attending high school in Denmark. The magnitude of the school-level variance of PA measures and how it is influenced by the inclusion of school characteristics is important for the development of school-based interventions aimed at increasing participation in PA and decreasing inactive behavioural patterns in young individuals. Girls of ethnic minorities, students with lower parental education and older students represent vulnerable groups with lower participation in several PA forms. Identification and circumventions of barriers complicating the participation in PA should be further identified in these groups.

## Conclusion

This study identified girls of perceived ethnic minority, students whose parents exhibit lower levels of education and older students as generally less likely to participate in several forms of PA during leisure time. However, the described groups did also have higher odds of participating in PA at school during leisure time. Underlined by the noteworthy school-level ICC of 9% for PA at school during leisure time, this implies that schools have the potential to activate these otherwise inactive groups of students by facilitating opportunities of PA at the schools. Also, school factors explained 8% of the variance in active transport to and from school, implying that schools have the potential to influence students’ behaviour in relation to active travel. Thus, high schools should facilitate and encourage activities such as leisure time PA within the framework of school and active transportation in all ages and vulnerable groups, as regular active commuting is an important contributor to overall PA levels [42, 43].

## Abbreviations

CI: Confidence Interval
DEGURBA: Degree of Urbanisation
HBSC: The Health Behaviour in School-aged Children study
ICC: Intra-class correlation
OR: Odds Ratio
PA: Physical activity

## References

[1] World Health Organization (2009). Global health risks: mortality and burden of disease attributable to selected major risks.: Department of Health Statistics and Informatics in the Information, Evidence and Research Cluster of the World Health Organization (WHO).

[2] Garber CE, Blissmer B, Deschenes MR (2011). American College of Sports Medicine position stand. Quantity and quality of exercise for developing and maintaining cardiorespiratory, musculoskeletal, and neuromotor fitness in apparently healthy adults: guidance for prescribing exercise. Med Sci Sports Exerc.

[3] Lee IM, Shiroma EJ, Lobelo F (2012). Effect of physical inactivity on major non-communicable diseases worldwide: an analysis of burden of disease and life expectancy. Lancet.

[4] Christensen AI, Davidsen M, Ekholm O (2014). Danskernes sundhed: Den nationale sundhedsprofil 2013.

[5] Hallal PC, Andersen LB, Bull FC (2012). Global physical activity levels: surveillance progress, pitfalls, and prospects. Lancet.

[6] Sawyer SM, Afifi RA, Bearinger LH (2012). Adolescence: a foundation for future health. Lancet.

[7] Viner RM, Ozer EM, Denny S (2012). Adolescence and the social determinants of health. Lancet.

[8] Currie C, Zanotti C, Morgan A (2012). Social determinants of health and well-being among young people. Health behaviour in school-aged children (HBSC) study: international report from the 2009/2010 survey.

[9] Azevedo MR, Araujo CL, Cozzensa da Silva M, Hallal PC (2007). Tracking of physical activity from adolescence to adulthood: a population-based study. Rev Saude Publica.

[10] Telama R, Yang X, Leskinen E (2014). Tracking of physical activity from early childhood through youth into adulthood. Med Sci Sports Exerc.

[11] Rasmussen M, Pedersen T, Due P (2015). Skolebørnsundersøgelsen 2014.

[12] Hjorth MF, Chaput JP, Michaelsen K (2013). Seasonal variation in objectively measured physical activity, sedentary time, cardio-respiratory fitness and sleep duration among 8-11 year-old Danish children: a repeated-measures study. BMC Public Health.

[13] Kristensen PL, Korsholm L, Moller NC (2008). Sources of variation in habitual physical activity of children and adolescents: the European youth heart study. Scand J Med Sci Sports.

[14] Health MX (2000). Outcomes of elementary school students in New Brunswick. The education perspective. Eval Rev.

[15] Maes L, Lievens J (2003). Can the school make a difference? A multilevel analysis of adolescent risk and health behaviour. Soc Sci Med.

[16] Murray DM, Catellier DJ, Hannan PJ (2004). School-level intraclass correlation for physical activity in adolescent girls. Med Sci Sports Exerc.

[17] Kristensen PL, Olesen LG, Ried-Larsen M (2013). Between-school variation in physical activity, aerobic fitness, and organized sports participation: a multi-level analysis. J Sports Sci.

[18] Hale DR, Patalay P, Fitzgerald-Yau N (2014). School-level variation in health outcomes in adolescence: analysis of three longitudinal studies in England. Prev Sci.

[19] Faulkner G, Zeglen L, Leatherdale S (2014). The relationship between school physical activity policy and objectively measured physical activity of elementary school students: a multilevel model analysis. Arch Public Health.

[20] Harvey A, Faulkner G, Giangregorio L, Leatherdale ST (2017). An Examination of school- and student-level characteristics associated with the likelihood of students' meeting the Canadian physical activity guidelines in the COMPASS study. Canadian journal of public health = Revue canadienne de sante publique.

[21] Hobin E, Leatherdale S, Manske S (2012). A multilevel examination of factors of the school environment and time spent in moderate to vigorous physical activity among a sample of secondary school students in grades 9-12 in Ontario, Canada. International journal of public health.

[22] Pisinger V, Mikkelsen SS, Bendtsen P (2017). The Danish National Youth Study 2014: study design, population characteristics and non-response analysis. Scand J Public Health.

[23] Ministry of Health. Bekendtgørelse af lov om videnskabsetisk behandling af sundhedsvidenskabelige forskningsprojekter. 2017 [cited 30 Jan 2018]; Available from: https://www.retsinformation.dk/Forms/R0710.aspx?id=192671

[24] Saltin B, Grimby G (1968). Physiological analysis of middle-aged and old former athletes. Comparison with still active athletes of the same ages. Circulation.

[25] Grimby G, Borjesson M, Jonsdottir IH (2015). The "Saltin-Grimby physical activity level scale" and its application to health research. Scand J Med Sci Sports.

[26] Statistics Denmark. Degree of urbanisation (DEGURBA) - Statistics Denmark vers 1.0. [cited 10 May 2017]; Available from: http://www.dst.dk/en/Statistik/dokumentation/nomenklaturer/degurba-%2D-danmarks-statistik

[27] Jensen VM, Rasmussen AW (2011). Danish education registers. Scand J Public Health.

[28] Pisinger V, Mikkelsen SS, Bendtsen P et al. The Danish National Youth Study 2014: study design, population characteristics, main findings and non-response analysis. *Accepted in* Scand J Public Health 2017. 10.1177/1403494817729283 28914164

[29] Nielsen G, Hermansen B, Bugge A (2013). Daily physical activity and sports participation among children from ethnic minorities in Denmark. Eur J Sport Sci.

[30] Owen CG, Nightingale CM, Rudnicka AR (2009). Ethnic and gender differences in physical activity levels among 9-10-year-old children of white European, south Asian and African-Caribbean origin: the child heart health study in England (CHASE study). Int J Epidemiol.

[31] Hebert JJ, Moller NC, Andersen LB, Wedderkopp N (2015). Organized sport participation is associated with higher levels of overall health-related physical activity in children (CHAMPS study-DK). PLoS One.

[32] Ostergaard L, Grontved A, Borrestad LA (2012). Cycling to school is associated with lower BMI and lower odds of being overweight or obese in a large population-based study of Danish adolescents. J Phys Act Health.

[33] Larsen K, Gilliland J, Hess P (2009). The influence of the physical environment and sociodemographic characteristics on children's mode of travel to and from school. Am J Public Health.

[34] Gropp KM, Pickett W, Janssen I (2012). Multi-level examination of correlates of active transportation to school among youth living within 1 mile of their school. Int J Behav Nutr Phys Act.

[35] Larouche R, Chaput JP, Leduc G (2014). A cross-sectional examination of socio-demographic and school-level correlates of children's school travel mode in Ottawa, Canada. BMC Public Health.

[36] Nelson NM, Foley E, O'Gorman DJ (2008). Active commuting to school: how far is too far?. Int J Behav Nutr Phys Act.

[37] Mandic S, Leon de la Barra S, Garcia Bengoechea E (2015). Personal, social and environmental correlates of active transport to school among adolescents in Otago, New Zealand. J Sci Med Sport.

[38] Schlossberg M, Greene J, Phillips PP (2006). School trips: effects of urban form and distance on travel mode. J Am Plan Assoc.

[39] Ministry of Education. Elevtal og fuldførelsesprocenter fordelt på gymnasial uddannelse. 2017 [cited 07 July 2017]; Available from: http://statweb.uni-c.dk/Databanken/uvmDataWeb/ShowReport.aspx?report=EAK-tilgang-gymudd

[40] Sallis JF, Saelens BE (2000). Assessment of physical activity by self-report: status, limitations, and future directions. Res Q Exerc Sport.

[41] Prince SA, Adamo KB, Hamel ME (2008). A comparison of direct versus self-report measures for assessing physical activity in adults: a systematic review. Int J Behav Nutr Phys Act.

[42] Sahlqvist S, Song Y, Ogilvie D (2012). Is active travel associated with greater physical activity? The contribution of commuting and non-commuting active travel to total physical activity in adults. Prev Med.

[43] Cooper AR, Andersen LB, Wedderkopp N (2005). Physical activity levels of children who walk, cycle, or are driven to school. Am J Prev Med.

